# Improved Sensitivity of Allergen Detection by Immunoaffinity LC-MS/MS Using Ovalbumin as a Case Study

**DOI:** 10.3390/foods10122932

**Published:** 2021-11-27

**Authors:** Martin Röder, Claudia Wiacek, Frauke Lankamp, Jonathan Kreyer, Wolfgang Weber, Elke Ueberham

**Affiliations:** 1 ifp Institut für Produktqualität GmbH, Wagner-Régeny-Str. 8, 12489 Berlin, Germany; roeder@produktqualitaet.com (M.R.); Lankamp@produktqualitaet.com (F.L.); weber@produktqualitaet.com (W.W.); 2 Institute of Food Hygiene, Leipzig University, An den Tierkliniken 1, 04103 Leipzig, Germany; claudia.wiacek@vetmed.uni-leipzig.de (C.W.); jonathan.kreyer@uni-leipzig.de (J.K.); 3 Fraunhofer Institute for Cell Therapy and Immunology (IZI), Perlickstr. 1, 04103 Leipzig, Germany

**Keywords:** egg allergen, monoclonal antibodies, allergen detection, processed food, mass spectrometry, Gal d 2

## Abstract

Food allergies are caused by severe hypersensitivity to specific food allergens such as the egg protein ovalbumin. It is therefore important to test food products for the presence of allergens to protect allergic people from accidental ingestion. For egg detection, ELISA is the only reasonable commercially available test format, although the recognition of target allergens can be affected by food processing, which may lead to false negative results. Current mass spectrometry-based detection methods may overcome this issue, but these approaches are often less sensitive. Here we combined the advantages of antibody-based and MS-based methods by developing an immunoaffinity LC-MS/MS technique to detect the common egg allergen Gal d 2. We investigated the principal functionality of this method with incurred cookie material containing whole egg powder. We found that the new method matched easily the sensitivity of egg specific ELISA tests. Further western blot experiments indicated that this strategy may be unaffected by food processing, providing an important alternative strategy for the detection and quantification of allergens in food.

## 1. Introduction

Food allergy is a potentially life-threatening immunological disorder caused by hypersensitivity to specific food allergens. There is currently no cure, so strict avoidance is required to prevent allergic reactions. Allergen analysis can identify and/or verify the presence of allergenic ingredients and unwanted (cross-contact) allergens in food throughout the production process from farm to fork, and is used by food suppliers, food producers, retailers and food safety agencies to ensure the availability of safe products for those with food allergies. Multiple analytical methods are available for the direct detection of allergenic food proteins, including the enzyme linked immunosorbent assay (ELISA), lateral flow devices (LFDs), and liquid chromatography tandem mass spectrometry (LC-MS/MS) [1,2]. Alternatively, the presence of allergens can be inferred indirectly by identifying the corresponding DNA sequence by real-time PCR [3]. Commercial ELISA and PCR kits are available for most important food allergens defined by current legislation. However, allergens from egg-white cannot be detected by PCR because egg white contains very little to no DNA [4].

Despite continuous improvements in detection methods, there is no universally superior technique and each of the methods listed above has unique advantages and disadvantages, creating issues with comparability across different assay formats [5,6]. A great advantage of antibody-based methods, regardless of which antibody is selected, is that detergents can be used during extraction, even with harsh procedures using 1–2% SDS with subsequent dilution to at least 0.05%. In contrast, most detergents interfere with LC-MS/MS analysis and extraction is therefore less efficient, hence the sensitivity of such methods remains unsatisfactory [7].

To combine the advantages of antibody-based and MS-based methods, we developed a proof-of-concept immunoaffinity LC-MS/MS technique using the common egg allergen Gal d 2 [8] as a case study. The selection of monoclonal antibodies for allergen detection is usually based on immunization with total protein extracts followed by screening without knowledge of the epitope sequence. In contrast, we screened for antibodies using peptides already known to be applicable in MS analysis. The resulting antibodies can be used for the affinity purification of allergens to improve the sensitivity of MS quantification [9].

We also investigated the treatment of egg proteins with heat (baking) and high hydrostatic pressure as examples of rigorous processing steps that influence both antigen–antibody interactions and peptide mapping [10,11]. Such processes cause the fundamental reorganization of protein structure by interfering with physical and chemical interactions such as Van der Waals forces and hydrogen bonds [12]. However, the covalent bonds that maintain the primary structure of linear epitopes and govern their interactions with peptide-specific antibodies should be preserved, facilitating detection by immunoaffinity clean-up and MS as discussed herein. Finally, we compared the performance of our new immunoaffinity method to a traditional ELISA for the detection of the egg allergen Gal d 2.

## 2. Materials and Methods

### 2.1. Selection of Suitable Tryptic Peptides for Gal d 2

Peptide selection was based on the 33 predicted trypsin cleavage sites in Gal d 2 matching the consensus (RK).[^P]. At least 12 of the resulting 34 peptides (Table 1) have been used by other authors to detect Gal d 2 by mass spectrometry based analytical approaches [13,14,15,16,17]. We also avoided peptides containing methionine, proline, those with multiple cleavage sites due to the presence of paired positively charged residues (RR, KK, KR or RK), and those with fewer than seven or more than 20 amino acids.

### 2.2. Generation of Monoclonal Antibodies

Mouse anti-Gal d 2 monoclonal antibodies were generated by immunizing female BALB/c mice (Janvier Labs, Le Genest-Saint-Isl, France) with ovalbumin (Sigma-Aldrich, Deisenhofen, Germany) denatured by incubating for 30 min at 60 °C in 8 M urea. The immunization experiments were approved by the State Animal Care and Use Committee (Landesdirektion Sachsen, Leipzig, Germany, V 07/14) and were carried out in accordance with the European Communities Council Directive (86/609/EEC) for the Care and Use of Laboratory Animals. Splenocytes were isolated from the mouse with the highest antibody titer specific for fully denatured ovalbumin containing both variants (ovalbumin and S-ovalbumin [18]) and were fused to X63.Ag8.653 myeloma cells (ACC 43; DSMZ, Braunschweig, Germany). Hybridoma supernatants diluted 1:50 in culture medium were screened by indirect ELISA on flat-bottom Nunc MaxiSorp 96-well ELISA plates (Thermo Fisher Scientific, Darmstadt, Germany) coated with fully denatured Gal d 2 (10 µg/mL) or biotinylated Gal d 2-specific peptides.

### 2.3. Screening Hybridoma Supernatants by Indirect Gal d 2 ELISA

Nunc Maxisorp plates were coated with denatured Gal d 2 (10 µg/mL), washed and sealed as previously described for legume antigens [19]. We added the hybridoma supernatants and incubated the plates for 30 min before washing three times with phosphate buffered saline (PBS, 154 mM NaCl) containing 0.05% Tween-20 (PBST) and adding the horseradish peroxidase (HRP)-conjugated goat anti-mouse IgG (Fc-specific) detection antibody (Dianova, Hamburg, Germany) for 20 min at room temperature. After another three washes as described above, we added the chromogenic substrate 3,3′,5,5′-tetramethylbenzidine (TMB-E; DUNN Labortechnik, Asbach, Germany). We then stopped the reaction by acidification with 0.05 M H_2_SO_4_ and measured the color intensity (OD at 450 nm) on a Sunrise ELISA plate reader (Tecan, Männedorf, Switzerland).

### 2.4. Screening Hybridoma Supernatants by Indirect Peptide ELISA

Biotinylated peptides were synthesized with N-terminal Biotin-Ahx groups by GenScript (Piscataway, NJ, USA). The peptides were diluted to 10 µg/mL in PBS and captured on streptavidin plates prepared by layering Nunc Maxisorp 96-well plates with 1 µg/mL streptavidin (Thermo Fisher Scientific) in carbonate buffer (pH 9.6) overnight at 4 °C. The coated plates were washed three times with PBST and blocked with Superblock reagent (Thermo Fisher Scientific) for 1 h at room temperature. The plates were then sealed using a liquid plate sealer (Candor BioScience, Wangen, Germany), air-dried, shrink-wrapped, and stored at room temperature. Following incubation with the biotinylated peptide overnight, the plates were washed three times with PBST and blocked with Superblock reagent as above. After removing the blocking reagent by tapping the plates, we added hybridoma culture supernatants and incubated the plates for 30 min. The plates were then washed three times as above and incubated with the HRP-conjugated anti-mouse IgG secondary antibody for 20 min at room temperature. The plates were washed again and incubated with the HRP substrate TMB-E to quantify the signal as described above.

### 2.5. Preparation and Characterization of Processed Material Containing Ovalbumin

We obtained four different commercially available whole egg powders: OVODAN Eiprodukte (Zeven, Germany), Würzteufel (Horb am Necka, Germany), OVOBEST Eiprodukte (Neukirschen-Vörden, Germany), and OVOPOL (Nowa Sól, Poland). We also obtained two egg white powders: OVOBEST and OVOPOL. The ovalbumin content was determined using a commercial ELISA kit (Morinaga Institute of Biological Science, Yokohama, Japan) following the extraction of 1 g egg powder using the manufacturer’s short extraction protocol.

For the high-pressure treatment of egg powder, we exposed 2.5 g OVODAN whole egg powder to 500 or 1000 MPa for 10 min at 20 °C in a high-pressure pilot plant (Dieckers, Willich, Germany) comprising two 25-mL cylindrical pressure chambers (Sitec, Maur, Switzerland) and a separate Ministat 240, 0–70 °C temperature control module (Peter Huber Kältemaschinenbau, Offenburg, Germany) to standardize the temperature in both pressure vessels.

Cookies were selected as a model bakery product food matrix for the incurred reference material. The ingredients were Diavita Type 405 wheat flour (Plange, Neuss, Germany), sugar (Südzucker, Mannheim, Germany), plant fat (OTHÜNA, Gera, Germany), baking powder (Ruf, Essen, Germany) and vanilla sugar (OSNA Nährmittel, Osnabrück, Germany). OVOPOL whole egg powder was used as the reference material for the production of egg-positive cookies. The plant fat and dry components were mixed for 45 min by smooth stirring in a KitchenAid artisan model (KitchenAid, Wilmington, DE, USA). Different allergen concentrations were achieved by adding a stock mixture containing 6711 mg/kg whole egg powder. The dough was portioned using a cookie press and baked at 175 °C in a convection oven (GGM Gastro, Ochtrup, Germany). After cooling, the mass loss of each cookie batch was measured, and the quantity of ovalbumin was determined by ELISA.

### 2.6. Western Blot

Proteins were separated by SDS-PAGE in precast gradient (4–15%) tris-glycine gels (Bio-Rad Laboratories, Munich, Germany) [20] and blotted onto PVDF membranes using iBlot 2 Transfer Stacks (Thermo Fisher Scientific). The membranes were blocked with 5% nonfat dried milk powder (Applichem, Darmstadt, Germany) in PBST (pH 8.0) and incubated overnight with the monoclonal antibodies at 4 °C. After washing, membranes were incubated with the HRP-conjugated goat anti-mouse IgG (Fc-specific) detection antibody for 1 h at room temperature.

### 2.7. Surface Plasmon Resonance (SPR) Spectroscopy

The kinetic properties of selected antibodies were determined using a Biacore T200 instrument (Cytiva, Schwerte, Germany). Monoclonal antibodies were aligned by capture on an in-house CM5 chip prepared using a mouse capture kit or a precoated protein G chip (Cytiva) with PBST as the dilution and running buffer. Kinetic binding constants were determined by injecting purified denatured Gal d 2 (ovalbumin) at a flow rate of 30 µL/min for 180 s. The surface was regenerated by pulsing for 60 s with 10 mM glycine/HCl (pH 1.7). Denatured Gal d 2 (10 mg/mL) was prepared by heating to 99 °C for 20 min in 2% SDS or to 60 °C in denaturing buffer containing 8 M urea, 2 M thiourea and 2% CHAPS [21]. Denatured Gal d 2 was diluted to the appropriate working concentration just before each run. Single cycle and multicycle assays were evaluated based on 1:1 binding using Biacore T200 Evaluation Software v3.2.

### 2.8. Coupling of Monoclonal Antibodies to the Affinity Matrix

Immunoaffinity columns were prepared by coupling 1 mg of the monoclonal antibody to 1 mL spherical pearl cellulose particles (Biotez, Berlin, Germany) overnight (~16 h) on a roller shaker. The next day, the affinity matrix was blocked, washed and stored in storage buffer at 4 °C. Before each test, 100 µL of the affinity matrix was added to an empty Chromabond polypropylene column (Macherey-Nagel, Düren, Germany) and sealed with the supplied filter elements.

### 2.9. Protein Extraction for Antibody Affinity Matrix Clean-Up

We transferred 3 g of homogenized sample material to a 50-mL Falcon tube and added 30 mL extraction buffer (10 mM ammonium penta-borate (Alfa Aesar, Karlsruhe, Germany), 16 mM Tris-HCl (Carl Roth, Karlsruhe, Germany) and 2.5 M urea, pH 8.5) at 20 °C. The sample was mixed and incubated in a water bath at 90 °C for 10 min with brief shaking after every 3 min. After cooling in tap water for 10 min, the samples were centrifuged at 4700× *g* for 30 min at 4 °C.

### 2.10. Tryptic Digestion of Proteins

We transferred 5 mL of the extraction supernatant to a 15-mL Falcon tube and added 5 mL 200 mM ammonium bicarbonate (VWR International, Leuven, Belgium) as a digestion buffer. The samples were reduced by adding 0.5 mL 200 mM dl-dithiothreitol (Sigma-Aldrich) and incubated at room temperature for 45 min, then alkylated by adding 0.5 mL 400 mM iodoacetamide (Sigma-Aldrich) in digestion buffer and incubated for 45 min in the dark at room temperature. We then added 0.5 mL trypsin (1 mg/mL in 50 mM acetic acid) and incubated the samples in a water bath at 37 °C for 1 h. Digestion was stopped by adding 360 µL 20% formic acid (Acros Organics, Geel, Belgium). The samples were stored at −20 °C. Prior to peptide clean-up, the samples were centrifuged at 4700× *g* for 20 min at 4 °C.

### 2.11. Immunoaffinity Clean-Up of Gal d 2 or Tryptic Gal d 2 Peptides

The ready-to-use immunoaffinity columns from Section 2.8 were washed twice with 3.3 mL binding buffer (20 mM sodium dihydrogen phosphate dihydrate, pH 7.0). We neutralized the tryptic digests of extracts by adding 10% (*v*/*v*) 1 M Tris pH 8.5 and added 3.3 mL of the neutralized peptide extract to each column at a flow rate of ~1 mL/min. The column was washed twice with 3.3 mL binding buffer and the peptides were eluted with 2 mL elution buffer (0.1 M glycine, pH 1.5).

### 2.12. Solid-Phase Extraction (SPE)

The peptides from the immunoaffinity clean-up step were purified by SPE on 60-mg C18 cartridges (Phenomenex, Aschaffenburg, Germany) to prepare them for HPLC. The columns were conditioned in 2 × 1 mL methanol (Chemsolute/Geyer, Renningen, Germany), then equilibrated in 2 × 1 mL 0.1% formic acid (FLUKA/Honeywell, Muskegon, MI, USA). The cartridge was loaded with peptides eluted from the affinity column (Section 2.11) and washed with 2 × 1 mL 0.1% formic acid. We added 5 µL DMSO (Chemsolute/Geyer) to each collector tube prior to elution with 2 × 0.375 mL methanol/0.1% formic acid (95:5 *v*/*v*). The eluted peptides were evaporated under flowing nitrogen at 40 °C and then dissolved in 75 µL 0.1% formic acid/acetonitrile (98:2 *v*/*v*). The samples were incubated at 4 °C for 30 min to promote solvation. Extracts were transferred to low-protein-binding tubes (Eppendorf, Hamburg, Germany), centrifuged at 15,000× *g* for 5 min at room temperature and transferred to HPLC glass vials for LC-MS/MS analysis.

### 2.13. LC-MS/MS

All HPLC instrument modules were from the 1290 Infinity or Infinity II lines (Agilent Technologies, Waldbronn, Germany), including the binary pumps (G7120A), multisampler (G7167B), flexcube (G4227A) and temperature-controlled column compartment (G7116B). We injected 10-µL samples and separated the peptides on a bioZen 2.6 µm peptide XB-C 18 column 2.1 × 100 mm (Phenomenex) at a flow rate of 0.3 mL/min. Solvent A was Mili-Q water (Merck, Darmstadt, Germany) containing 0.1% formic acid, and solvent B was acetonitrile plus 0.1% formic acid. The following gradient was applied over a time of 15 min 20 s: 0–0.87 min, 98% A; 0.87–7.33 min, 98–60% A; 7.33–8.33 min, 60–2% A; 8.33–11.6 min, 2% A; 11.6–11.73 min, 2–98% A, 11.73–15.33 min, 98% A.

Peptide ions were detected using a QTRAP 6500+ triple quadrupole MS system (Sciex, Darmstadt, Germany) in positive electrospray mode with the following settings: curtain gas flow = 45 L/min, collision gas = high, source temperature = 450 °C, ion spray voltage = 5.5 kV, ion source gas 1 = 62 L/min, ion source gas 2 = 35 L/min. Tryptic peptides were used for the optimization of multiple reaction monitoring (MRM) parameters: de-clustering potential (DP), collision energy (CE), and cell exit potential (CXP). Therefore, we used the syringe pump injection mode. Automatic optimization was achieved using the Compound Optimization feature of the MS-control software Analyst v1.7.1 (Sciex). The detailed MRM method for the detection of ovalbumin is shown in Table 2. The entrance potential was maintained at 10 V. The chromatograms were interpreted using Sciex OS v17.0 (Sciex).

## 3. Results

### 3.1. Selection of Peptides and Corresponding Peptide Specific Monoclonal Antibodies

**Figure 1:**
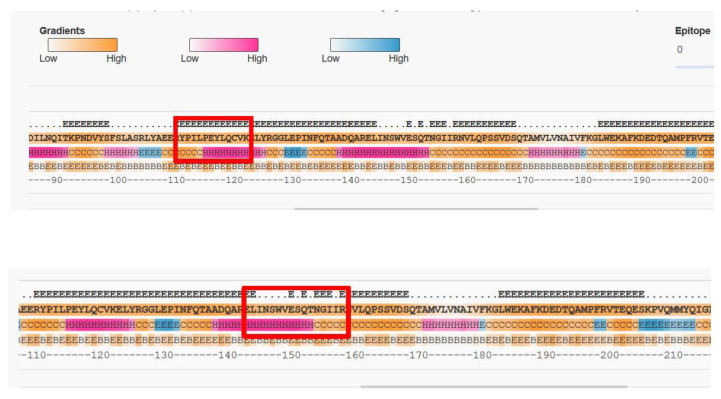
Prediction of Gal d 2 peptide epitopes using BepiPred 2.0. The Gal d 2 sequence was analyzed to identify the accessible (E = exposed) and inaccessible (B = buried) amino acids. Red squares show peptides with no binding antibodies among the 585 clones we tested. The color gradients show the probability of secondary structures (pink = helices, blue = sheets and orange = coils).

Selected monoclonal antibodies were produced on mg scale by culturing hybridoma and then purifying the culture supernatants over protein G affinity columns. Overall we isolated 585 individual clones that bound specifically to denatured Gal d 2, and stored them as cryopreserves for further analysis. The threshold for selection was an OD_450nm_ > 0.1 in an indirect ELISA using diluted hybridoma supernatant (1:50 *v*/*v*). This OD indicates the presence of high-affinity antibodies at low concentrations and/or medium-affinity antibodies at moderate concentrations. We then screened the clones using the emphasized in bold text MS-compatible peptides of Table 1 which fulfilled the selection criteria denoted in Section 2.1. All of these highlighted peptides have been verified by NCBI PROTEIN BLAST to be unique for ovalbumin from hen’s egg except for YPILPEYLQCVK which is characteristic for ovalbumin from bird eggs in general. All peptides have been used in previous studies [13,14,15,16,17]. We identified unique clones specific for peptides ADHPFLFCIK, DILNQITKPNDVYSFSLASR and GGLEPINFQTAADQAR, respectively, as well as 29 clones specific for peptide HIATNAVLFFGR and 41 clones specific for peptide ISQAVHAAHAEINEAGR (all bold in Table 1). In contrast, screening with the peptides YPILPEYLQCVK and ELINSWVESQTNGIIR (bold black in Table 1) yielded no positive clones with specific binding activity. Although we identified multiple antibodies that bound to specific Gal d 2 peptides, we recovered none that were specific for YPILPEYLQCVK and ELINSWVESQTNGIIR. One potential explanation is the lack of immunogenicity, although BepiPred 2.0 (which indicates the exposed amino acids within a folded protein) predicted the full accessibility of YPILPEYLQCVK and at least partial accessibility of ELINSWVESQTNGIIR (Figure 1). Alternatively, the presentation of the peptides to the monoclonal antibody during the indirect ELISA may have been disrupted by the streptavidin-biotin coupling or partial misfolding of the synthetic peptide.

### 3.2. Western Blot

**Figure 2:**
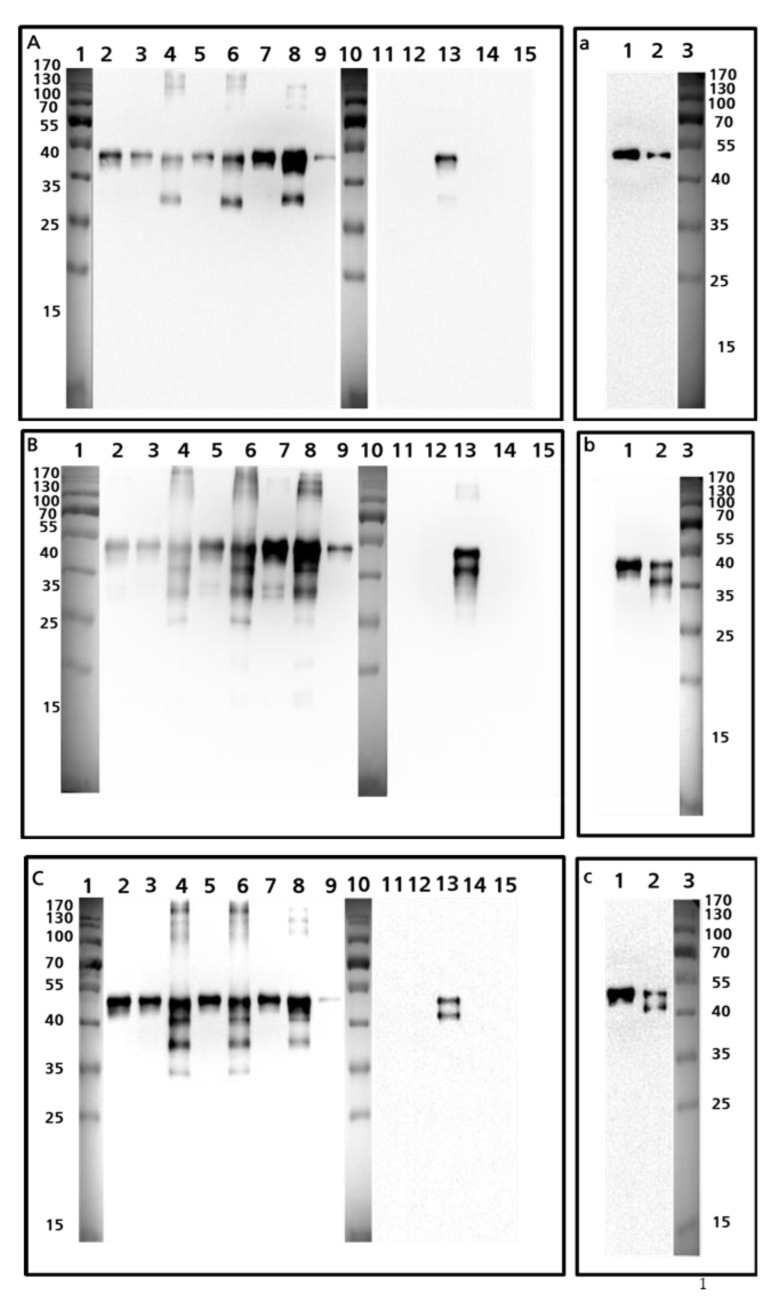

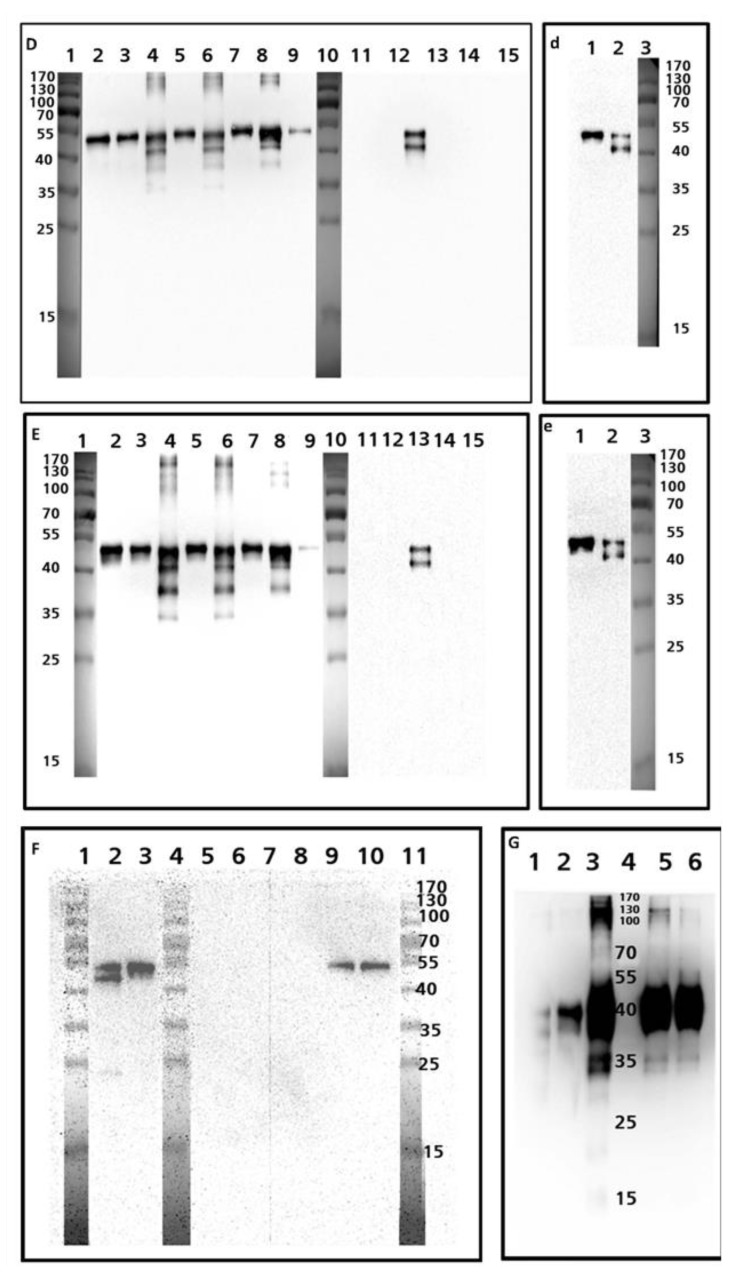
Western blot of various egg preparations and cookies with the peptide-specific monoclonal anti Gal d 2 antibodies. (**A**,**a**) Representative western blot of egg preparations probed with HIATNAVLFFGR-specific monoclonal antibodies. (**B**,**b**) Representative western blot of egg preparations probed with probed with ISQAVHAAHAEINEAGR-specific monoclonal antibodies. (**C**,**c**) Representative western blot of egg preparations probed with GGLEPINFQTAADQAR-specific monoclonal antibodies. (**D**,**d**) Representative western blot of egg preparations probed with ADHPFCIK-specific monoclonal antibodies. (**E**,**e**) Representative western blot of egg preparations probed with DILNQITKPNDVYSFSLASR-specific monoclonal antibodies. Lane numbers refer to the following preparations: 1 = size markers; 2 = OVODAN whole egg; 3 = Würzteufel whole egg; 4 = Würzteufel egg white; 5 = OVOBEST whole egg; 6 = OVOBEST egg white; 7 = OVOPOL whole egg; 8 = OVOPOL egg white; 9 = chicken egg yolk (Sigma-Aldrich); 10 = size markers; 11 = conalbumin from chicken egg white, substantially iron free (Sigma-Aldrich); 12 = conalbumin from chicken egg white (Sigma-Aldrich); 13 = albumin from chicken egg white (Sigma-Aldrich); 14 = trypsin inhibitor from chicken egg white null (Sigma-Aldrich), 15 = trypsin inhibitor from chicken egg white (Sigma-Aldrich). Small membranes labeled with lower-case letters (a, b, c, d, e) show the banding pattern on the two forms of ovalbumin: 1 = ovalbumin; 2 = S-ovalbumin; 3 = size markers. (**F**) Representative western blot of commercial ovalbumin preparations probed with the DILNQITKPNDVYSFSLASR-specific monoclonal antibody. 1 = size markers; 2 = preparation containing S-ovalbumin; 3 = preparation without S-ovalbumin; 4 = size markers; 5–8 = cookies without egg; 9 = cookies with 5 ppm egg; 10 = cookies with 20 ppm egg. (**G**) Representative western blot of commercial ovalbumin preparations and unprocessed or processed egg powders probed with a complete mixture of all peptide-specific antibodies. 1 = preparation containing S-ovalbumin; 2 = preparation without S-ovalbumin; 3 = both preparations unprocessed; 4 = size markers; 5 = both preparations treated at 500 MPa for 10 min at 20 °C in a high-pressure pilot plant; 6 = both preparations treated as above but at 1000 MPa.

Western blots probed with the peptide-specific antibodies revealed different banding patterns for the various whole egg preparations (Figure 2, capital letters). Blots probed with antibodies specific for the peptide HIATNAVLFFGR (Figure 2A,a) showed a single band in pure preparations of ovalbumin, regardless of which preparation is considered, either the preparation not containing S-ovalbumin (Figure 2a, lane 1) or the one containing S-Ovalbumin (Figure 2a, lane 2). In contrast, the banding patterns were almost identical in all blots probed with antibodies recognizing the peptides ISQAVHAAHAEINEAGR (Figure 2B,b), GGLEPINFQTAADQAR (Figure 2C,c), ADHPFLFCIK (Figure 2D,d), and DILNQITKPNDVYSFSLASR (Figure 2E,e). In the purified ovalbumin preparation containing S-ovalbumin (Sigma-Aldrich), a unique band with a molecular weight of 45 kDa was detected and the resulting double band pattern in the preparation that was indicated to contain both forms (SIGMA-Aldrich) was interpreted to represent the ovalbumin and S-ovalbumin proteins (Figure 2b–e). S-ovalbumin becomes more abundant during the storage of unfertilized eggs due to the change in pH resulting from the loss of CO_2_ [18] (Figure 2, lower case letters). This pH change leads to both deamidation of asparagine and a conformational shift, resulting in higher hydrophobicity of S-ovalbumin compared to ovalbumin [18]. Altered charge and hydrophobicity can lead to altered SDS loading of proteins and a shift in electrophoretic mobility [22,23]. The antibodies generated against the linear peptide sequences do recognize these epitopes in processed food as shown in Figure 2F (incurred cookies) and Figure 2G (high pressure processed egg preparation).

### 3.3. Characterization of Monoclonal Antibodies by SPR Spectroscopy

**Figure 3:**
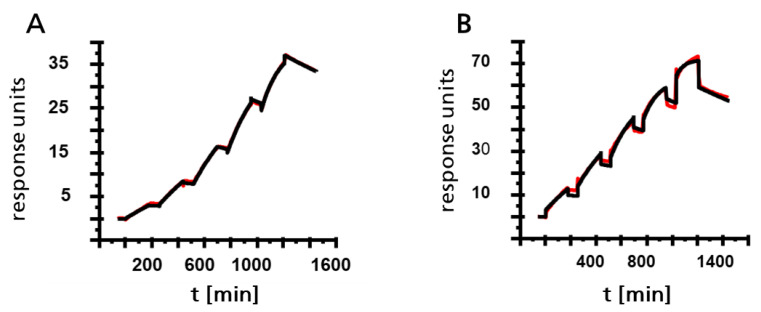
Characterization of monoclonal antibodies by SPR spectroscopy analysis using single cycle kinetics. (**A**) Representative plot of an antibody specific for peptide ISQAVHAAHAEINEAGR and the analyte Gal d 2 in a MS-compatible denaturing buffer containing urea (KD = 1.438 × 10^−8^) (**B**) Representative plot of the same antibody with the analyte Gal d 2 denatured in SDS-containing buffer (KD = 6.37 × 10^−9^). Red = raw data and black = fitted curve for 1:1 binding.

The kinetic parameters of the antibodies were determined by SPR spectroscopy using purified Gal d 2 as the analyte. All tested antibodies showed nanomolar affinity for the allergen (Figure 3). As expected, the pre-treatment of Gal d 2 with a buffer containing detergents to mimic typical extraction conditions caused a degree of protein unfolding and thus increased access to the epitopes. The best results were obtained by denaturation at 95 °C in 50 mM Tris containing 2% SDS, just before each measurement. In single-cycle injections, K_D_ values were up to an order of magnitude higher when using denaturation solutions containing SDS rather than urea (Figure 3).

### 3.4. Commercial Sandwich ELISA (Morinaga)

Table 3 summarizes the concentration of ovalbumin after mass loss during baking as quantified by ELISA. Starting from a whole egg powder premix, the above concentrations were added to the cookie dough, and the calculated concentrations of ovalbumin were obtained in the processed cookies. Up to 86% of the incorporated ovalbumin could be quantified by ELISA in the processed cookie matrix, but at the lowest concentration the value fell to 35%.

### 3.5. Immuno-Affinity LC-MS/MS Analysis (Clean-Up after Tryptic Digestion)

**Figure 4:**
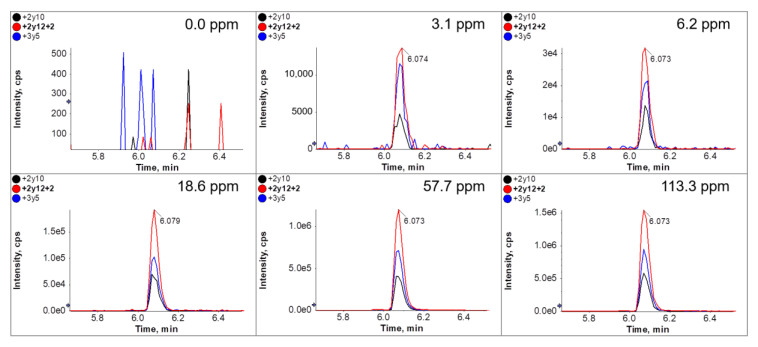
XIC-Chromatograms with 3 MRM transitions from peptide GGLEPINFQTAADQAR in the analyzed incurred cookie materials. The panels show the MRM transitions of three selected fragments (+2y10—black; +2y12 + 2—red; +3y5—blue) from peptide GGLEPINFQTAADQAR in each sample. The corresponding ovalbumin concentration is shown in the top right-hand corner. From each sample, we used 3.3 mL of neutralized extract for immunoaffinity clean-up and solid-phase extraction prior to LC-MS/MS.

**Figure 5:**
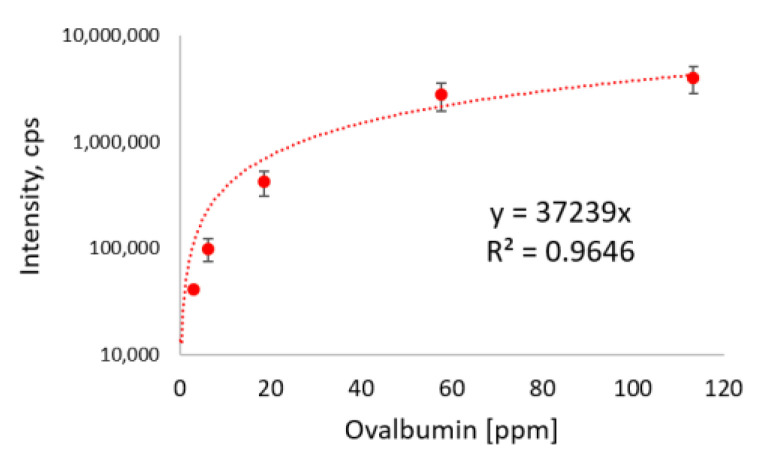
Regression of area intensities (*n* = 3) from transition +2y12 + 2 of peptide GGLEPINFQTAADQAR of the six ovalbumin concentrations in analyzed incurred cookie materials shown in Figure 4.

We coupled the antibodies to cellulose beads without the optimization of coupling ratios or reaction times. We were able to load 3.3 mL of a neutralized tryptic digested egg white protein extract at concentrations of up to 1% without breakthrough on the affinity column, so this volume was chosen for most of the subsequent experiments. The most sensitive results were achieved using the antibody specific for peptide GGLEPINFQTAADQAR. The MS data obtained with SPE-purified peptides following immunoaffinity clean-up of the cookie material revealed distinct peaks with high signal-to-noise (S/N) ratios at all analyte concentrations (Figure 4). The S/N ratio for peptide GGLEPINFQTAADQAR 2y12 + 2 was 133.1 at the lowest concentration of 3.1 mg/kg. These proof-of-principle experiments also showed that the MS signal intensity correlated to a certain degree with the ovalbumin concentration in the cookies, suggesting that the data were also at least partially quantitative. Figure 5 shows the regression curve for the most intense transition 2y12 + 2 of peptide GGLEPINFQTAADQAR. Each sample was extracted three times and different batches of immunoaffinity columns were used.

**Figure 6:**
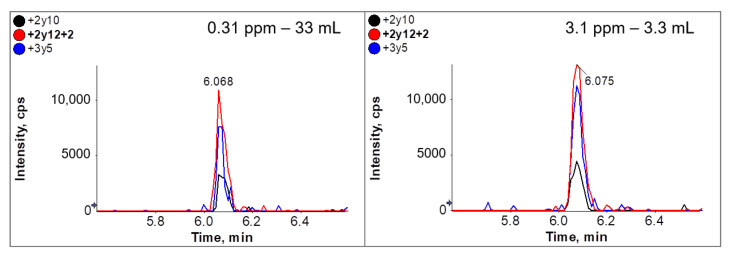
XIC-Chromatograms with 3 MRM transitions from peptide GGLEPINFQTAADQAR from mimicked cookie material with 0.31 mg/kg (33 mL used for purification) (left) vs. real cookie material 3.1 mg/kg (3.3 mL used for purification). The cookie extract with the lowest ovalbumin concentration of 3.1 mg/kg was diluted 10-fold with ovalbumin-free extract to mimic an extract with an ovalbumin concentration of 0.31 mg/kg. We applied the immunoaffinity cleanup and solid-phase extraction process to 33 mL of the diluted extract (0.31 mg/kg ovalbumin) and 3.3 mL of the undiluted extract. The chromatograms show the three MRM transitions of peptide GGLEPINFQTAADQAR (+2y10—black; +2y12 + 2—red; +3y5—blue).

To mimic very low ovalbumin concentrations, the digested sample extract from the cookie containing 3.1 mg/kg ovalbumin was diluted 1:10 with the digested sample extract of the cookie lacking ovalbumin, reducing the concentration to 0.31 mg/kg. This is below the detection threshold of normal LC-MS/MS. However, by using a 10-fold higher sample volume during affinity clean-up (33 mL), a clear MS signal was observed, comparable to that achieved by the analysis of the 3.1 mg/kg sample (Figure 6).

**Figure 7:**
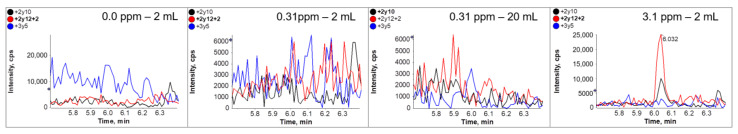
LC-MS/MS analysis of cookie materials containing 0, 0.31 and 3.1 mg/kg ovalbumin (see Table 3). The cookie extract with the lowest ovalbumin concentration of 3.1 mg/kg was diluted 10-fold with ovalbumin-free extract to mimic an extract with an ovalbumin concentration of 0.31 mg/kg. The samples were extracted and cleaned up by conventional solid-phase extraction alone. We used 2 mL of the 0, 0.31 and 3.1 mg/kg samples and, on one occasion, 20 mL of the 0.31 mg/kg samples prior to LC-MS/MS. Extracted ion chromatograms representing peptide GGLEPINFQTAADQAR are shown with three transitions (+2y10—black; +2y12 + 2—red; +3y5—blue).

The results from “conventional SPE” LC-MS/MS experiments without immunoaffinity clean-up using different volumes are shown in Figure 7. In contrast to immunoaffinity clean up, the use of higher volumes did not improve the signal intensity when samples with ovalbumin concentration below the limit of detection (approx. 2 ppm) were used. Figure 7 shows that the signal obtained with the diluted 0.31 ppm sample was almost identical in the regular SPE approach with 2 mL and a ten-fold increased approach with 20 mL, although the peptide amount should be comparable to a sample which contained 3.1 ppm. However, using the immunoaffinity approach a 10 fold increase of sample volume of the 0.31 ppm sample lead to a 10 fold increased signal, which was nearly equivalent to the 3.1 ppm sample. Further experiments will be necessary to optimize peptide-specific immunoaffinity enrichment in general and particularly in the context of different matrices.

### 3.6. Immuno-Affinity LC-MS/MS Analysis (Clean-Up after Extraction)

Using the immunoaffinity approach we expected a second set up to be possible: the direct enrichment of the undigested Gal d 2. This should be functional since the selection of the antibodies itself was based on their binding to the undigested protein. Unfortunately, we could not confirm this experimentally. Using antibody coupled affinity matrix as well as uncoupled matrix material as a control sample, we observed comparable high intensities for Gal d 2 peptides. Thus, we assume detection of the nonspecific binding of proteins to the affinity matrix itself and not only to the antibodies. The reason for this non-specific binding is unclear within the current setup, but the observation described suggests that it may be connected with the column material itself, or less likely the affinity matrix. The column and antibody-coupled affinity matrix are identical to the one we used for peptide purification, where we successfully demonstrated enrichment and purification of digested proteins. In this setup, we did not observe MS-specific signals for other peptides from egg proteins. This indicates the specificity of the antibodies used and the functionality of the peptide clean-up strategy. Even if there had been non-specific binding of proteins, it would be irrelevant and undetectable in this set-up, as there is no additional tryptic digest that releases the specific peptide masses used in the MS. It will be investigated in further experiments whether e.g., an adjustment of the buffer compositions, the antibody concentration on the column, the washing steps between binding and elution or even a pre-blocking of the column can prevent non-specific binding.

## 4. Discussion

Allergen analysis typically involves the antibody-based detection of proteins in food using methods such as sandwich or competitive ELISA, bead assays, immunoaffinity chromatography (LFAs), dip-stick assays or antibody-based SPR spectroscopy [24]. These methods are dependent on the antibody quality [25,26,27], especially properties such as affinity and specificity. Antibodies that recognize non-linear epitopes are occasionally found to bind with higher affinity than those recognizing linear epitopes [28]. ELISAs are generally sensitive and specific, but food processing often destroys native protein structures, preventing the detection of allergens with non-linear epitopes [29,30,31]. ELISAs are also used for the quantitative detection of food allergens [32], but the OD readout must be transformed into mg/mL or mg/kg using a conversion factor which is based on internal calibration [33]. Different calibrators are used by the suppliers of different ELISA kits, so the results across different assays are difficult to compare. PCR-based assays can be used instead to detect the genes encoding particular allergens, but this approach is not suitable for egg allergens due to the absence of sufficient quantities of DNA in egg white.

Another method for the detection of food allergens is MS, typically LC-MS/MS. This is based on the detection of tryptic peptides that are unique to the target allergen, and stable isotope labeled (SIL) peptides can in principle determine the absolute quantity of allergens in a sample. MS has a very high specificity as long as the peptide is allergen-specific. Unlike antibody-based methods, there is no risk of cross-reactivity in MS. However, LC-MS/MS often lacks the sensitivity of ELISA and is rarely offered as an alternative for the detection of food allergens.

In this study, we developed a method that combines immunoaffinity clean-up and MS to exploit the best aspects of antibody-based and MS-based detection methods, using ovalbumin as a case study. This combination of technologies can overcome the disadvantages of both ELISA and MS, as previously reported [9]. Both ELISA and LC-MS/MS begin with the solubilization of proteins to extract the allergen from the complex food matrix. Our SPR data showed that the affinity of the peptide-specific antibodies (expressed as K_D_) increases by an order of magnitude when using an extraction buffer containing SDS rather than urea, the latter being more compatible with current MS-based methods [34,35,36].

Our LC-MS/MS method for the detection of Gal d 2 is part of a more complex method for egg detection that is used for routine analysis in the IFP contract food testing facility, and has recently been accredited as a qualitative method by the German DAkks according to DIN EN ISO 17025. Unlike ELISA methods, the sensitivity of MS declines as the protein content of the matrix increases, varying between 5 and 100 mg/kg whole egg powder (equivalent to 2–40 mg/kg ovalbumin). Even with peptide enrichment by SPE, MS therefore does not approach the sensitivity of most ELISAs. However, given that ELISAs often cannot detect allergens in processed foods, MS may still detect the presence of egg proteins when ELISA does not.

We demonstrated the suitability of immunoaffinity LC-MS/MS in a series of peptide enrichment experiments, offering the potential to overcome multiple difficulties encountered when using MS alone. By using the presented immunoaffinity clean-up approach, the sensitivity of the method seems to be no longer limited, because the loading volume of the columns can be adjusted. Thus, the detection limits for all allergen parameters can be easily shifted to very low concentrations, which should be suitable to satisfactorily analyze allergens in very large portion sizes and especially in foods with the claim “free from (allergen)”.

However, we found that only one of two possible immunoaffinity enrichment strategies is currently suitable: the enrichment of specific peptides after tryptic digestion, which generates almost pure peptide mixtures that can be detected by MS. The absence of >90% of competing peptide fragments and interfering metabolites/byproducts results in a very high S/N ratio and thus improves sensitivity while preventing contamination in the MS instrument and the associated cleaning and maintenance costs. Although the alternative strategy (enriching peptide-associated whole proteins) was not successful, there are several potential benefits such as the removal of most irrelevant proteins (reducing digestion times and improving the S/N ratio) and allowing the analysis of several marker peptides from the same allergen. Further studies are required to determine whether protein enrichment is possible and whether protein or peptide enrichment confers the most advantages overall. The immunoaffinity method requires the cost-effective production of monoclonal antibodies that are conjugated with high precision for use in immunoaffinity columns, and the ability of hybridomas to meet this challenge needs to be addressed in detail.

The diverse western blot profiles of the different antibodies also require further discussion. It is unclear why monoclonal antibodies that recognize the epitope HIATNAVLFFGR were unable to bind S-ovalbumin (Figure 2B, lane 13 and Figure 2b, lane 2) because this is a thermostabilized conformational version of Gal d 2 and not a partial digest of ovalbumin from the C-terminus, hence the HIATNAVLFFGR sequence should still be available. However, the strong denaturing buffer (2% SDS, 0.5% 2-mercaptoethanol) would be expected to unfold the protein completely. Even so, antibodies targeting all peptides except HIATNAVLFFGR recognized both forms of ovalbumin on the western blots.

It is useful to quantify S-ovalbumin as well as ovalbumin degradation products because these affect the functionality and processability of foods containing egg protein—for example, gels from egg white preparations containing S-ovalbumin are known to have a low gel strength [18]. Accordingly, our panel of antibodies could be used not only as affinity purification tools for MS, but also for in-process quality control during the industrial manufacture of egg preparations to monitor aging (transformation into S-ovalbumin).

The higher affinity achieved by denaturing Gal d 2 just before SPR analysis confirms that the antibody-based pre-purification of peptide mixtures is beneficial for MS quantification. The denaturation step, involving heat and exposure to the detergent SDS, makes the epitopes fully accessible and promotes the solubilization of allergens during extraction from complex food matrices, although this step would be incompatible with MS analysis without pre-purification.

## Figures and Tables

**Table 1:** Peptides generated by the complete digestion of Gal d 2 using the serine protease trypsin.

Position of Cleavage Site	Peptide Sequence	Peptide Length [aa]	Peptide Mass [kDa]
17	MGSIGAASMEFCFDVFK	17	1840.157
20	ELK	3	388.464
47	VHHANENIFYCPIAIMSALAMVYLGAK	27	2977.55
51	DSTR	4	477.475
56	TQINK	5	602.688
59	VVR	3	372.468
62	FDK	3	408.455
85	LPGFGDSIEAQCGTSVNVHSSLR	23	2374.61
105	**DILNQITKPNDVYSFSLASR**	20	2281.55
111	LYAEER	6	779.848
123	**YPILPEYLQCVK**	12	1465.771
127	ELYR	4	579.654
143	**GGLEPINFQTAADQAR**	16	1687.829
159	**ELINSWVESQTNGIIR**	16	1859.069
182	NVLQPSSVDSQTAMVLVNAIVFK	23	2460.871
187	GLWEK	5	631.729
190	AFK	3	364.445
200	DEDTQAMPFR	10	1209.296
219	VTEQESKPVQMMYQIGLFR	19	2284.674
227	VASMASEK	8	821.944
229	MK	2	277.382
264	ILELPFASGTMSMLVLLPDEVSGLEQLESIINFEK	35	3864.521
277	LTEWTSSNVMEER	13	1581.717
278	K	1	146.189
280	IK	2	259.349
285	VYLPR	5	646.787
287	MK	2	277.382
291	MEEK	4	535.613
323	YNLTSVLMAMGITDVFSSSANLSGISSAESLK	32	3294.736
340	**ISQAVHAAHAEINEAGR**	17	1773.926
360	EVVGSAEAGVDAASVSEEFR	20	2009.114
370	**ADHPFLFCIK**	10	1190.425
382	**HIATNAVLFFGR**	12	1345.567
386	CVSP	4	404.482

The peptides in bold met the selection criteria that predicted their suitability for LC-MS/MS analysis.

**Table 2:** Parameters of the scheduled MRM method (MRM detection window 120 s, DP = de-clustering potential, CE = collision energy, CXP = cell exit potential).

Q1 (*m/z*)	(Q3 *m/z*)	RT (min)	Marker Peptide	DP (V)	CE (V)	CXP (V)
761.1	930.5	6.85	Gal d 2 DILNQITKPNDVYSFSLASR. + 3y8	141	45	26
761.1	767.4	6.85	Gal d 2 DILNQITKPNDVYSFSLASR. + 3y7	141	31	22
930.0	1116.6	6.93	Gal d 2 ELINSWVESQTNGIIR. + 2y10	161	45	32
620.3	673.4	6.93	Gal d 2 ELINSWVESQTNGIIR. + 3y6	141	25	20
620.3	572.4	6.93	Gal d 2 ELINSWVESQTNGIIR. + 3y5	171	25	42
844.4	1121.5	6.12	Gal d 2 GGLEPINFQTAADQAR. + 2y10	156	43	34
844.4	666.3	6.12	Gal d 2 GGLEPINFQTAADQAR. + 2y12 + 2	151	35	20
563.3	560.3	6.12	Gal d 2 GGLEPINFQTAADQAR. + 3y5	76	17	40
761.9	1036.5	6.74	Gal d 2 YPILPEYLQCVK. + 2y8	151	35	30
761.9	518.8	6.74	Gal d 2 YPILPEYLQCVK. + 2y8 + 2	151	29	30
673.4	1024.6	6.24	Gal d2 HIATNAVLFFGR.2y9	146	35	28
673.4	923.5	6.24	Gal d2 HIATNAVLFFGR. + 2y8	146	35	26
673.4	809.5	6.20	Gal d2 HIATNAVLFFGR. + 2y7	16	37	26
791.4	1052.5	5.75	Gal d2 LTEWTSSNVMEER 2y9	146	37	32
791.4	951.4	5.75	Gal d2 LTEWTSSNVMEER 2y8	141	37	28
887.5	1138.6	4.42	Gal d2 ISQAVHAAHAEINEAGR. + 2y11	151	59	38
887.5	1067.5	4.42	Gal d2 ISQAVHAAHAEINEAGR. + 2y10	151	53	36
887.5	996.5	4.42	Gal d2 ISQAVHAAHAEINEAGR. + 2y9	161	53	32
624.3	924.5	6.10	Gal d2 ADHPFLFCIK. + 2y7	61	39	30
624.3	827.4	6.10	Gal d2 ADHPFLFCIK. + 2y6	71	35	28
624.3	324.1	6.10	Gal d2 ADHPFLFCIK. + 2b3	11	29	10
624.3	681.3	6.10	Gal d2 ADHPFLFCIK. + 2b6	66	35	24

**Table 3:** Various model cookies spiked with whole egg powder quantified by ELISA.

Premix Added to Dough * [g/kg]	Calculated ppm Ovalbumin in Dough **	Quantified ppm Ovalbumin by ELISA in Cookies
0	-	0
1.7	3.1	1.1 ± 0.01
3.3	6.2	2.6 ± 0.12
10.0	18.6	11.7 ± 0.32
31.0	57.7	49.6 ± 2.19
61.8	113.3	89.6 ± 2.75

* Premix with 6711 ppm whole egg powder containing 1645 ppm ovalbumin; ** corrected by mass loss.

## Data Availability

Not applicable.

## Abbreviations

ELISA—enzyme-linked immunosorbent assay, SDS-PAGE—sodium dodecyl-sulfate polyacrylamide electrophoresis, LC-MS/MS—liquid chromatography coupled with tandem mass spectrometry, PBS—phosphate-buffered saline, TMB-E—3,3′,5,5′-tetramethylbenzidine ELISA substrate.
